# Biotechnological advances and applications of human pluripotent stem cell-derived heart models

**DOI:** 10.3389/fbioe.2023.1214431

**Published:** 2023-07-25

**Authors:** Priyadharshni Muniyandi, Colin O’Hern, Mirel Adrian Popa, Aitor Aguirre

**Affiliations:** ^1^ Institute for Quantitative Health Science and Engineering, Division of Developmental and Stem Cell Biology, Michigan State University, East Lansing, MI, United States; ^2^ Department of Biomedical Engineering, College of Engineering, Michigan State University, East Lansing, MI, United States; ^3^ Institute of Cellular Biology and Pathology Nicolae Simionescu, Bucharest, Romania

**Keywords:** cardiotoxicity, human pluripotent stem cells, cardiac, organoid, engineered heart tissues, drug

## Abstract

In recent years, significant biotechnological advancements have been made in engineering human cardiac tissues and organ-like models. This field of research is crucial for both basic and translational research due to cardiovascular disease being the leading cause of death in the developed world. Additionally, drug-associated cardiotoxicity poses a major challenge for drug development in the pharmaceutical and biotechnological industries. Progress in three-dimensional cell culture and microfluidic devices has enabled the generation of human cardiac models that faithfully recapitulate key aspects of human physiology. In this review, we will discuss 3D pluripotent stem cell (PSC)-models of the human heart, such as engineered heart tissues and organoids, and their applications in disease modeling and drug screening.

## Introduction

Cardiovascular diseases (CVDs) are a set of conditions that significantly affect the function and structure of the heart (e.g., coronary heart disease, myocardial infarction, hypertension, congestive heart failure, cardiomyopathies, congenital defects), and constitute the leading cause of death worldwide (GBD Viewpoint Collaborators, 2020; Tsao et al., 2022). CVDs often stem from vascular dysfunction, like atherosclerosis, thrombosis, or high blood pressure, which then leads to heart pathology and dysfunction (Basson, 2008). However, a less publicized, but equally significant source of CVDs is drug-induced cardiotoxicity, which is a side effect induced by exposure to therapeutic drugs (e.g., chemotherapeutics, psychotropics) or candidate therapeutic drugs in development (Onakpoya et al., 2016; Mladěnka et al., 2018). Cardiotoxicity is a substantial concern for patients, physicians, and pharmaceutical companies. For example, ventricular arrhythmias are a common manifestation of chemotherapy induced cardiomyopathy (CICM) cardiotoxicity (Stone et al., 2021), resulting in 28% of all drug withdrawals from the US market (Gwathmey et al., 2009). Furthermore, some drug candidates that pass preclinical trials can still be cardiotoxic and unsafe for patients, and cardiac monitoring may be later recommended, indicating that standard preclinical models do not fully recapitulate important aspects of human physiology, particularly cardiac electrophysiology. A highly utilized preclinical model to test for drug induced cardiotoxicity evaluates seven cardiac ion channels referred to as the comprehensive *in vitro* proarrhythmia assay (Crumb et al., 2016) and reveals the importance of the human ether-a-go-gorelated gene (hERG) channel as the most frequently blocked in an evaluation of 30 clinical drugs. The CiPA study was done on the cloned equivalent of human channels in non-human CHO or human (embryonic kidney cells) cell lines overexpressing the hERG potassium ion channel. However, these non-cardiac cell lines cannot come close to recapitulating the detailed characteristics of heart cells, tissues, structure, and function. These differences and the approval of cardiotoxic drugs underscores a need for representative human heart models to test and screen for cardiotoxicity during pharmacotherapy development.

Over the past 2 decades, advances in human pluripotent stem cell technologies (hPSCs) -a term which refers to both embryonic stem cells (ESCs) and induced pluripotent stem cells (iPSCs)- have allowed for the generation of patient-specific pluripotent stem cells (Takahashi and Yamanaka, 2006), supplying a promising platform for cell therapy and drug testing *in vitro* (Wang et al., 2021b), and biobanking of patient-specific hPSCs has created a pathway toward a precision medicine future (Huang et al., 2019). During this same period, two dimensional (2D) hPSC-derived cardiomyocyte (CM) models have emerged (Burridge et al., 2012), providing a human heart tissue platform for cardiotoxicity screening (Blinova et al., 2017) and drug discovery (Mercola et al., 2013). Continued advancements in hPSC differentiation protocols and tissue engineering by many groups is allowing for improved CMs that more closely reproduce the metabolic and functional characteristics of *in vivo* CMs necessary for industrial biomedical applications (Denning et al., 2016). More recent protocols have generated atrial and ventricular specific CMs (Kleinsorge and Cyganek, 2020). Indeed, CMs are easy to generate and have such a foothold in the pharmaceutical industry that more complex human heart tissue models will need to demonstrate functional niches (Oren et al., 2021).

In recent years, advances in human PSC-derived organ-like models have emerged as powerful alternatives to overcome the limitations of current cardiotoxicity models (Thomas et al., 2021). Although pre-clinical studies of anti-cancer drug induced cardiotoxicity in 2D hPSC-CM monolayers show promise (Schwach et al., 2020), the 2D CM monolayers still lack critical structural characteristics of the native heart and do not feature other relevant cell types in the human heart that would benefit from organoid models (Cho et al., 2022; Ramirez-Calderon et al., 2022). To overcome this limitation, 3D hPSC-derived heart organoid and gastruloid models are being produced with high physiological relevance when compared to their native counterparts. The integration of bioengineering and bioscaffolds with CMs to generate 3D tissue/organ models and applications to CVD discovery (Sacchetto et al., 2020) along with organoid technology (Lancaster and Knoblich, 2014) open up new opportunities to study CVD origins and evaluate drugs. Thus, advancements in 3D hPSC-derived heart models will be critical for developing non-toxic and effective pharmaceutical therapies. This review will characterize some of the current methods for generating 3D hPSC-derived heart models and how they’ve been applied for disease modeling and drug/cardiotoxicity screening.

## Engineering 3D hPSC-derived heart models

**FIGURE 1:**
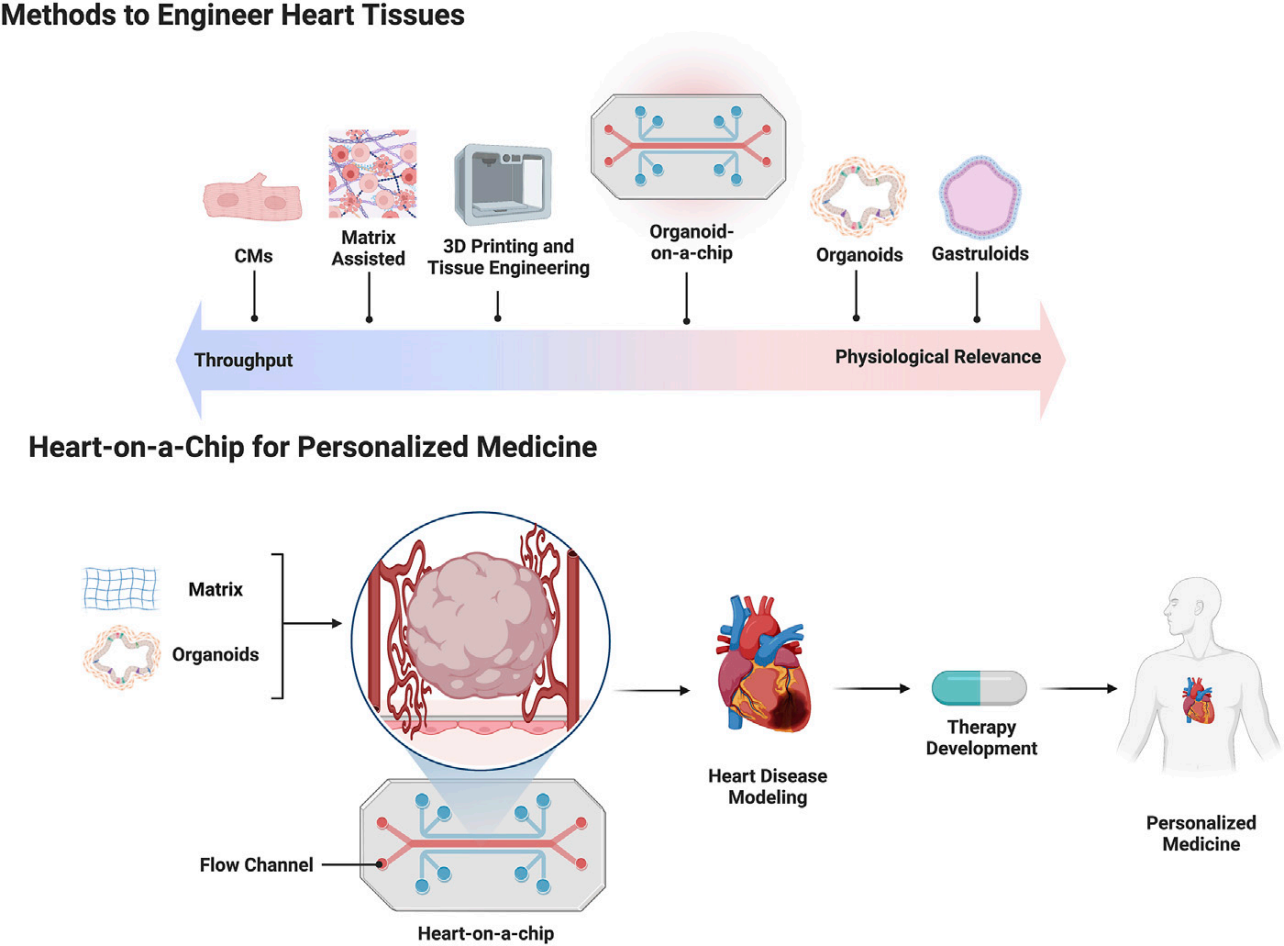
The top half of the figure is a schematic depicting current methods used to engineer human heart tissue placed against a linear spectrum comparing throughput capability versus physiologic relevance. The bottom half of the figure depicts a flow chart outlining the use of a heart-on-a-chip platform for making patient specific cardiac organoids for personalized medicine applications.

Organ development arises through dynamic and complex interactions between early embryonic progenitors during gastrulation (Ghimire et al., 2021). The heart is the first functional organ to develop, however, the early stages of cardiogenesis are still not fully understood (Muñoz-Chápuli and Pérez-Pomares, 2010). One bottleneck in cardiogenesis research has been the lack of developmentally relevant *in vitro* models for the rapid testing of drugs and mechanisms. Since the discovery and widespread use of hPSC technology, early cardiac models have focused primarily on 2D hPSC-CM monolayers and their differentiation (Protze et al., 2019). Many of these approaches focus on generating hPSC-derived heart tissue to study cell specifications during development, CM maturation, contractility, disease, and drug testing (Stein et al., 2021). Despite these advancements, there are still significant challenges in modeling key processes of early cardiogenesis with *in vitro* 2D and 3D human heart models based on hPSCs (Lancaster and Huch, 2019; Schutgens and Clevers, 2020). There is a need to engineer more complex and faithful 3D hPSC-heart models, and several methods have emerged over the past years (Liu et al., 2022). These methods include bottom-up approaches based on cellular self-assembly and directed-assembly approaches that utilize multicell-type seeding on exogenous extracellular matrices or 3D bioprinting. Additionally, organ/organoid-on-a-chip technologies use elements of both approaches, providing longer term viability and increased complexity (Takebe et al., 2017). Each strategy has its own advantages and limitations in terms of biological complexity, drug and clinical applicability, and scalability (Figure 1). The following sections elaborate on stem cell models of gastruloids, spheroids and organoids, and engineered heart tissues (EHTs) and current use in drug studies.

*Gastruloids.* In the mid 20th century, there were a few theories as to how cell specification occurred during embryogenesis. In 1952, Turing hypothesized that the interaction between two chemical substances, which he referred to as morphogens, could give rise to self-organizing patterns in a developing organism, termed reaction-diffusion responses (Turing, 1952). In 1969, Wolpert proposed that cells receive positional information from their environment, typically in the form of concentration gradients of signaling molecules, which instruct them to adopt specific fates and differentiate into particular cell types (Wolpert, 1969). Recently, a review suggested that mechanochemical feedback between tissue shape and cell fate, termed crosstalk, governs tissue self-organization (Shahbazi, 2020). These are the driving theories of cell specification during embryogenesis and PSCs have played a critical role in understanding the mechanisms driving early embryogenesis. Gastrulation is a crucial part of embryogenesis that occurs after implantation of a fertilized egg in the uterine endometrium and formation of a blastocyst (Shahbazi, 2020). The epiblast, arising from the inner cell mass of the blastocyst, undergoes epithelial-to-mesenchymal transition, ultimately forming three primitive germ layers: ectoderm, mesoderm, and endoderm (Tam and Behringer, 1997). This process is mediated by mechanical cues and positional signaling pathway gradients along different axes of the developing embryo. Many groups are recapitulating aspects of embryogenesis of organs *ex vivo* in hPSC organoid and gastruloid technologies (Simunovic and Brivanlou, 2017). This includes early gastruloid models with regions expressing cardiogenic and heart field markers (Rossi et al., 2020) as well as complex multi-chambered and innervated human hearts (Olmsted and Paluh, 2022). Schultheiss *et al.* (1997) revealed that bone morphogenic protein 4 (BMP4) was required for myocardial differentiation during early embryonic development in ESCs. Peerani *et al.* (2007) later demonstrated that hPSCs possess the ability to self-organize, responding to specific signals secreted within their microenvironment in a position-dependent manner. This phenomenon was also observed when the cells were cultured on micropatterned surfaces, which have now been extensively engineered to enhance the spatial distribution of cells (Warmflash et al., 2014; Blin, 2021). Minn *et al.* (2021) developed a 2D human gastruloid protocol using hPSCs cultured on a micropatterned surface in the presence of BMP4 to successfully differentiate germ layer, extraembryonic, and primordial germ-like cells validated by single-cell RNA sequencing, which unveiled the presence of sub-populations of mesodermal cells, particularly cardiogenic cells. It is important to note that micropatterned surfaces confine cells to a 2D space, while embryonic development occurs in a 3D environment. The self-organizing properties of hPSCs need not require scaffolds. In 2020, Moris *et al.* (2020) were the first to showcase an hPSC-derived 3D gastruloid when exposed to CHIR9901, a potent WNT-activator, that could aggregate and elongate along a specific axis within a few hours. Gene expression analysis revealed the first and second heart fields in the anterior region. Wnt activation is also applied in the elongating multilineage organized cardiac (EMLOC) advanced heart gastruloid. All-in-all, the current novel state of cardiac organ gastruloids in mice (Rossi et al., 2020) and humans (Olmsted and Paluh, 2022) are largely focused on heart development and congenital heart defects and cardiotoxicity screening of drugs has not yet been explored.

*Cardiac spheroids and organoids.* Spheroids are small, simple, and scaffold-free 3D aggregates of differentiated stem cells or primary adult cells that develop through cell-to-cell interactions (Scalise et al., 2021). They typically consist of multiple cell types that self-assemble but do not exhibit the cellular organization, distinct tissue architecture, or functional capacity found in organs. Organoids, on the other hand, are 3D structures derived from PSCs, progenitors, and/or differentiated cells that self-organize through cell-cell and cell-matrix interactions to recapitulate aspects of native tissue architecture and function *in vitro* (Marsee et al., 2021). Organoids have at least one organ sub-function and a degree of lineage-dependent spatial organization, making them more structurally and functionally complex than spheroids, and better at mimicking the native microenvironment. Generating spheroids and organoids requires only a few manual steps and minimal cell counts, typically ranging from 1,000 to 10,000 cells per specimen, allowing for high-throughput characterization of multiple replicates in an array format (Scalise et al., 2021). Although cardiac spheroids have shown to be useful in drugs screening and cardiotoxicity studies (Polonchuk et al., 2017; Christoffersson et al., 2018; 2019), cardiac organoids are a more promising platform for studying developmental biology, modeling disease, drug screening, studying the effects of environmental stressors, and elucidating intercellular crosstalk mechanisms (Miyamoto et al., 2021). Within the last decade, many groups published human heart organoid models, reviewed in Zhao *et al.* (2021). Silva *et al.* (2021) described cardiac organoids with endodermal-derived tissues (e.g., gut and intestine) and showed how the emergence of gut tissue contributes to cardiac maturation. Drakhlis *et al.* (2021) proved the role of the foregut endoderm in cardiac organoids by inducing the WNT pathway to mimic the architectural characteristics of the native heart anlagen before heart tube formation. Regarding cardiac organoids derived from one germ layer, Hofbauer *et al.* (2021) were able to generate cardiac organoids by modulating WNT activation and activin A concentrations in the absence of extracellular matrix (ECM) proteins. They showed that mesodermal cells alone could form chamber-like cavities, which were otherwise impaired by BMP signaling inhibition or HAND1 knockout hPSC lines, resulting in reduced NKx2.5 levels in the cardiac mesoderm. They further demonstrated that culturing epicardial tissue with cardiac organoids led to epicardium expansion, migration, and differentiation, similar to observations *in vivo* (Simões and Riley, 2018). Tan *et al.* (2021) also reported the influence of epicardium on myocardium expansion in a 3D cardiac microtissue. In our laboratory, we developed a method to induce mesoderm-derived cardiac and epicardial cells in self-assembled heart organoids using three sequential time-specific Wnt modulation steps in suspension-cultured embryoid bodies (Lewis-Israeli et al., 2021). Transcriptomic analysis revealed that our human heart organoids closely resembled fetal heart tissue and produced multiple cardiac-specific cell lineages, including CMs, cardiac fibroblasts, endocardial cells, and endothelial cells. These organoids exhibited well-defined sarcomeres, mitochondria, gap junctions, and tubular structures such as T-tubules. They also displayed robust beating with well-defined action potential waves, including QRS complexes, T waves, and P waves, as detected using a multi-electrode array. Overall, the generation of human heart organoids based on cellular self-assembly shows promise in producing complex human heart tissues that require minimal maintenance and have high throughput ability. Such advanced organoids are suitable for addressing complex metabolic disorders associated with pathological conditions, such as congenital heart defects, including those induced by pregestational diabetes (Lewis-Israeli et al., 2022; Kostina et al., 2023). In another study, we also produced self-assembled organoids with anterior-posterior patterning from an endogenous retinoic acid gradient originating at the atrial pole, where proepicardial and atrial populations reside, thereby mimicking the developmental process of the primitive heart tube (Volmert et al., 2022). One of the current limitations of cardiac organoids is that the tissue they model is immature compared to native adult human heart tissue (Zhao et al., 2021). Developing more mature cardiac organoids would be important in the context of drug and cardiotoxicity screening since many of the FDA-approved drugs that are cardiotoxic are typically administered in adulthood due to chronic conditions. Furthermore, organoids often have misassembled regions which may cause slight variations between individual organoids that must be considered in high throughput analysis. Organoids are not trilineage in cellular origin (endoderm, mesoderm, ectoderm) and so do not model certain phases of embryonic development, such as the pre-implanted blastocyst (Rivron et al., 2018), post-implantation development (Shao et al., 2017a; 2017b; Harrison et al., 2017; Sozen et al., 2018; Zheng et al., 2019), and gastrulation (Van Den Brink et al., 2014; Beccari et al., 2018). However they are useful in modeling aspects of organ function and can be combined in assembloids to expand functions (Vogt, 2021). Despite these limitations, organoid technology holds great potential for revolutionizing the fields of medicine and drug development.

*Engineered heart tissues.* To generate EHTs with greater complexity, ECM architecture and biochemistry are tailored to provide instructive microenvironments that promote tissue growth, organization, and maturation (Baker and Chen, 2012; Aljohani et al., 2018; Lou and Mooney, 2022). These matrices and scaffolds aim to mimic the physical, chemical, and mechanical properties of natural ECMs and can include natural and synthetic polymers (Frantz et al., 2010; Kyburz and Anseth, 2015). Some matrix-assisted approaches used for the generation of EHTs are described below.

*Hydrogels* are hydrophilic polymer 3D networks capable of absorbing fluids (Lou and Mooney, 2022). They are ideal for cell encapsulation and seeding due to their permeability and swelling characteristics. The exceptional biocompatibility and diffusion properties of hydrogels render them appealing for use in cardiac tissue engineering (CTE) applications (Saludas et al., 2017). Hydrogel scaffolds can be tailored to achieve desired architectural configurations, with cellular uniformity maintained within the scaffolds. Although natural hydrogels like collagen, gelatin, chitosan, hyaluronic acid, fibrin, and Matrigel exhibit high biocompatibility, bioactivity, and biodegradability, they possess limited mechanical properties. This limitation can be overcome by combining hydrogels with physical cues to alter the differentiation PSCs to a desired cell type (Tortorella et al., 2022). Various studies have investigated the inclusion of bioactive molecules and growth factors within hydrogels to promote angiogenesis (Leslie-Barbick et al., 2011; Leslie-Barbick et al., 2011; Prakash Parthiban et al., 2017), cell proliferation (Schneider et al., 2019), migration (Topman et al., 2013), and differentiation (Benoit et al., 2008). For example, Wimmer *et al* (2019) demonstrated the application of matrix-assisted organoid fabrication in producing complex capillary networks consisting of endothelial cells, pericytes, and basement membranes. The use of 3D matrices such as collagen and Matrigel enabled vascular differentiation, resulting in engineered microvessels possessing hollow lumens that successfully integrated into the host vasculature upon *in vivo* transplantation. Overall, hydrogels possess exceptional biocompatibility and diffusion properties that make them an appealing option for engineering more complex 3D-hPSC derived heart models.

*Decellularized extracellular matrices* (dECMs) are biologically derived extracellular matrices obtained by removing cellular components from native tissues, supplying an endogenous platform for tissue engineering and regenerative medicine applications (Saldin et al., 2017; Nguyen et al., 2018; Zhang et al., 2022). dECMs have been generated from human cadaveric hearts and reseeded onto whole heart decellularized cardiac scaffolds (Guyette et al., 2016). Even though dECMs provide a native ECM, the low mechanical stability of the dECM limits its utility in bioprinting applications (Brown et al., 2022; Isaeva et al., 2022). To overcome this limitation, a recent study used gelatin methacryloyl and methacrylated hyaluronic acid hydrogels in combination with dECMs to create cardiac tissue-like constructs with tunable mechanical properties (Basara et al., 2021). However, challenges remain with dECMs, including the need for thorough cell removal and preservation of structural integrity, which can hinder subsequent cell reseeding and lead sample variation (Zhang et al., 2022). Moreover, the decellularization process is often laborious and time-consuming.

*3D bioprinting* is another approach that offers a distinct, top-down take to CTE, enabling arbitrary control over macro-scale tissue architecture and cellular distribution (Dey and Ozbolat, 2020). Advanced scaffold-free 3D bioprinting has been investigated for direct deposition of cell aggregates. This technique has successfully generated various cardiac tissue constructs, such as heart valves, blood vessels, and myocardium (Arai et al., 2018; Liu et al., 2021). Researchers at the University of Minnesota recently have 3D-printed the first ever centimeter sized heart organoids (Kupfer et al., 2020). The process involved a specialized bio-ink, made from ECM proteins and human stem cells, printed onto ventricular structures. The corresponding stem cells were expanded to a high cell density on the ventricular structures and differentiated into CMs that contracted synchronously. While their printed cardiac muscle models demonstrated promising results in small kinetic models, this is insufficient for large scale animal/human models with thicker myocardial walls and higher oxygen perfusion demands; therefore, further development of this technology is needed. Work is currently being done to address this limitation by using bioprinting compatible hydrogel inks that enables the incorporation of a sacrificial thermo-responsive support hydrogel directly into an intact organ (Wang et al., 2021a). This approach has been employed in attempts to generate a human heart. Ideally, this platform would enable rapid construction of perfusable patient-specific tissue with shape and volume up to therapeutic standards, opening exciting avenues previously unexplored by traditional CTE methods.

*Microfluidic chip platforms.* Despite the favorable characteristics of 3D organoids for *in vitro* disease modeling and drug screening, there are certain limitations, such as small-scale production, lack of automation, cost, and reproducibility, that jeopardize their translation to the pharmaceutical industry (Clevers, 2016). Some organoid applications are done in static culture, which hinders oxygen and nutrient perfusion to cells within the core of the organoid. To address this limitation, an interesting concept has emerged: integrating organoids with advanced microphysiological systems based on microfluidic technology (Park et al., 2019; Leung et al., 2022). Microfluidic chips supply a controlled environment that ensures optimal temperature, pH, nutrients, oxygen supply, and waste removal through continuous flow of fresh culture medium (Park et al., 2019). Sensors and actuators allow for precise control and monitoring of microscale variables that mimic *in vivo* conditions during organoid culture. Many microfluidic organ-on-a-chip platforms have been developed using primary cells to create a variety of biomimetic organ models, such as lung (Huh et al., 2010), liver (Nakao et al., 2011), kidney (Jang et al., 2013), heart (Ma et al., 2015; An et al., 2016), gut (Kim et al., 2012), vessel (Franco and Gerhardt, 2012), skin (Atac et al., 2013), bone marrow (Torisawa et al., 2014), and blood-brain barrier (Griep et al., 2013). Thus, integrating hPSC-derived organoids with microfluidic platforms, also known as organoid-on-a-chip, can better mimic patient specific physiological conditions than organoids alone because they can emulate perfusion, mechanical, and other parameters crucial for tissue and organ physiology (Garreta et al., 2021). Advancements in organoid-on-a-chip technology could be used to create more faithful 3D heart tissue models, that would benefit drug discovery (Figure 1) (Takebe et al., 2017). Furthermore, organoid-on-a-chip platforms would allow for the integration of multiple tissue compartments to simulate multiple organs and make systematic pharmacokinetic predictions for new drugs (Leung et al., 2022). In this context, the pharmaceutical industry estimated a significant reduction in drug development cost by adopting microphysiological system technologies which could also replace animal models by providing better control over biochemical parameters to generate physiologically relevant tissue (Ingber, 2022). Limitations of the heart organoid*-*on*-*a*-*chip technology include a lack of high throughput scalability, increased costs, and potential limited complexity of tissues. However, with time, heart organoids-on-a-chip are expected to improve and to be a promising tool for disease modeling and pharmaceutical drug/toxicity screening.

## Disease modeling, drug safety assessment, and cardiotoxicity screening

*Disease Modeling*. 3D hPSC-derived heart models can aid in the study and treatment of rare diseases or CVDs. For example, heart organoids have been used to model acute myocardial infarction (AMI), which displayed tissue regeneration after injury (Arhontoulis et al., 2022; Gania et al., 2022; Paz-Artigas et al., 2023). Even heart spheroids mimicking scarred tissue after an AMI have been made by assembling CMs and fibroblasts to specific ratios to create larger cardiac tissues with structural features (Takeda et al., 2021; Li et al., 2022). Our lab developed self-assembling hPSC-derived heart organoids cultured in high glucose to study the effects of pre-gestational diabetes on cardiac development, which resulted in larger organoids, arrhythmias, and decreased oxygen consumption (Lewis-Israeli et al., 2021; Lewis-Israeli et al., 2022). Furthermore, there are many genetic conditions that can be modeled using 3D hPSC-derived cardiac organ-like models. For example, human heart organoid models of Barth syndrome, a mitochondrial disorder caused by a Tafazzin gene mutation that leads to dilated cardiomyopathy, have been generated using patient-derived iPSCs and microchips (Wang et al., 2014). Heart organoids have been used to explore the connections between metabolism and transcription regulation in protein kinase adenosine monophosphate-activated non-catalytic subunit gamma 2 (PRKAG2) cardiomyopathy caused by missense mutations in the PRKAG2 regulatory subunit (Ntusi et al., 2016; Manhas et al., 2022; Novelli et al., 2022; Vuckovic et al., 2022). Regarding modeling hypertrophic cardiomyopathy, CMs generated from titin-mutated iPSCs replicated cardiomyopathic pathology and showed how inadequate sarcomere formation leads to impaired tissue responses to stress (Schick et al., 2018; de Boer et al., 2022). Recent advancements in gene editing technology, such as CRISPR/Cas9, have proven their utility in organoids for disease modeling and treatment. This includes rescuing diseased patient iPSCs or generating disease-related mutations in healthy iPSCs (Platt et al., 2014; Abudayyeh et al., 2016). While the former has not yet been attempted in organoid models, it has been successfully performed in animal models with Duchenne’s muscular dystrophy (DMD) via exon skipping. This involved removing mutated exons from the dystrophin gene, resulting in a shortened but functional dystrophin protein that relieved DMD symptoms (Kuhr et al., 2007; McGreevy et al., 2015; Zaynitdinova et al., 2021). In DMD mice and dogs, improvements in skeletal and heart muscle were noted with local applications or intravenous injections. A similar approach was used in a transgenic pig model lacking exon 52 of the dystrophin allele using a split Cas9 version (Platt et al., 2014; Abudayyeh et al., 2016; Chemello et al., 2021; Echigoya et al., 2021). Regarding generating disease models with CRISPR/Cas9 technology, Hofbauer *et al.* (2021) used CRISPR gene editing to model cardiac malformations by knocking out HAND1 and NKX2.5 in an hPSC line, while Drakhlis *et al.* (2021) created cardiac organoids using an NKX2.5 knockout hPSC line and observed reduced tissue compaction, reduced adhesion between cardiomyocytes, and cardiomyocyte hypertrophy, similar to the uncompacted trabecular overgrowth and hypertrophy seen in NKX2.5 knockout mice. Overall, the use of 3D hPSC-derived heart organ-like platforms has proven to be a valuable tool for modeling CVDs.

*Drug Screening.* The process of drug discovery involves multiple steps, initiating with target selection, such as a protein or a pathway, that is relevant to the disease of interest (Han et al., 2018). Phenotypic screening or target-based screening is performed to identify molecules able to modulate the target, termed leads. These top leads are then optimized by medicinal chemistry to enhance their selectivity, activity, and stability. In this stage, the compounds are mainly tested *in vitro* using cell lines and/or primary cells to measure their effects. The best compounds are then moved to *in vivo* animal studies to evaluate toxicity, optimal dose, and administration method. After synthesis, a compound undergoes extensive preclinical testing to ensure safety and efficacy before entering clinical trials (Dahlin et al., 2015).

A broad classification of each of these compounds may have a wide range of effects and uses. These compounds can be classified into the following pharmacological categories summarized in Table 1.

**TABLE 1:** A list of drug classifications and their general clinical applications and effects.

DRUG classification	Applications and effects
**Beta Blockers**	Reduce heart rate, decrease blood pressure, and limit heart muscle contraction. They are often used in the treatment of hypertension, heart failure, and certain arrhythmias.
**Antiarrhythmics**	Prevent or treat irregular heartbeats or arrhythmias.
**Calcium Channel Blockers**	Block calcium from entering cells of the heart and blood vessel walls, resulting in lower blood pressure. Verapamil and diltiazem are also classified as antiarrhythmics.
**Non-steroidal anti-inflammatory drugs (NSAIDs)**	Relieve pain and inflammation.
**Antineoplastics**	Used in the treatment of cancer.
**Beta-Adrenergic Agonists**	Mimic the effects of endogenous epinephrine and norepinephrine and are often used in the treatment of heart failure, hypertension, and asthma.
**Potassium Channel Blockers**	Inhibit the actions of potassium channels and are often used to treat arrhythmias.
**Phosphodiesterase Inhibitors**	Block phosphodiesterases, which break down cAMP and cGMP. They are used to treat conditions like erectile dysfunction and pulmonary hypertension.
**Angiotensin II Receptor Blockers (ARBs)**	Block the effects of angiotensin II, a hormone that constricts blood vessels. They are often used in the treatment of hypertension and heart failure.
**Antibiotics/Antivirals**	Used in the treatment of various bacterial and viral infections.
**Antipsychotics/Antidepressants**	Used to treat various mental health disorders.
**Miscellaneous**	There are several compounds that do not fit neatly into one category, or their primary use is not primarily cardiovascular, including compounds like Rosiglitazone (an antidiabetic drug), Tamoxifen (used in breast cancer treatment), Buspirone (an anxiolytic), Ranolazine (anti-angina drug), Minoxidil (a vasodilator).

**FIGURE 2:**
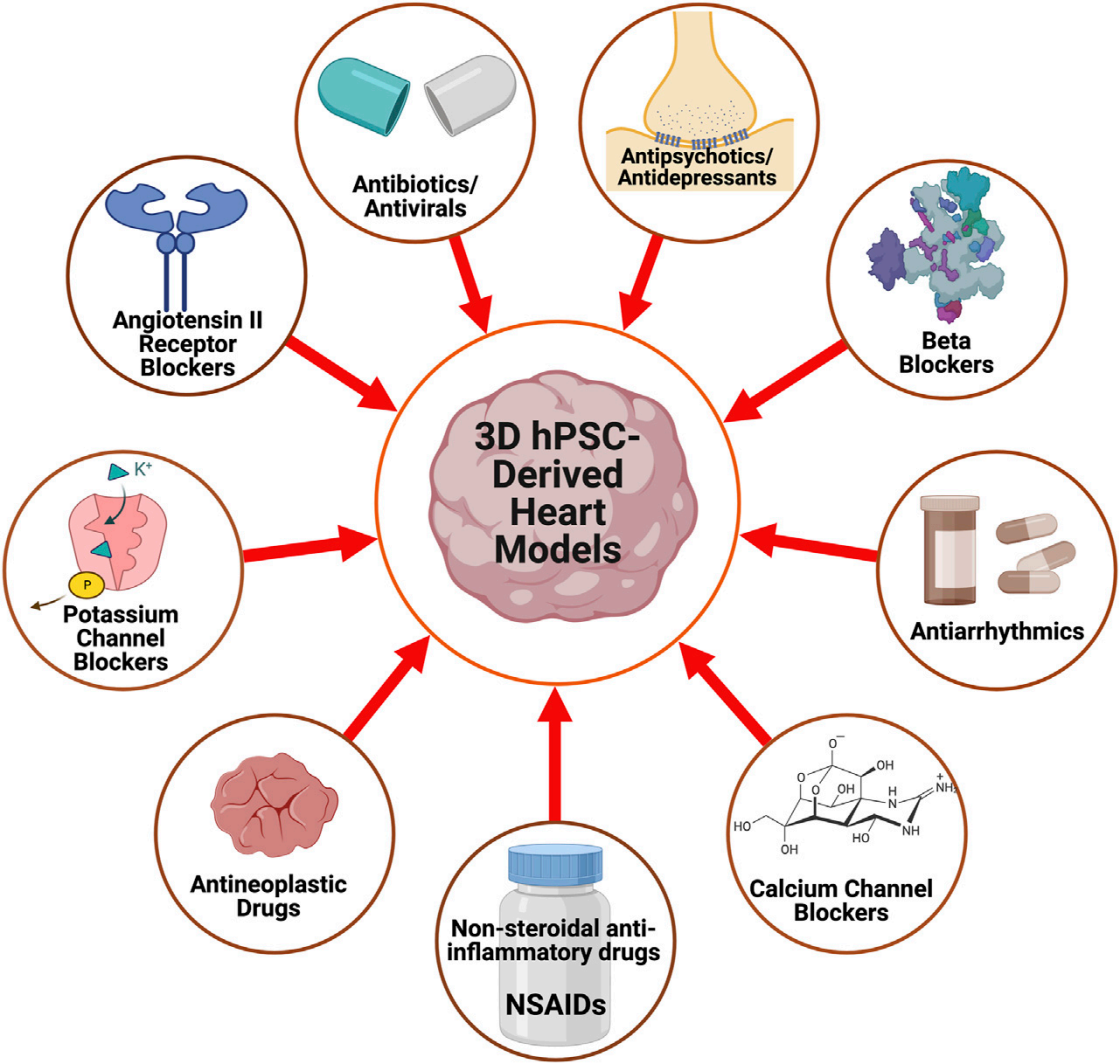
A schematic displaying the pharmacological agents that have been utilized to study and characterize 3D hPSC-derived heart models.

With advances in CTE, *in vitro* drug screenings with 3D hPSC-human heart tissue can help assess drug efficacy for heart conditions and analyze their impact on tissue function (Heydari et al., 2021; Rao et al., 2022). Here, we highlight some of the drug screening and cardiotoxicity studies that have been done on 3D human heart tissue models *in vitro* (Figure 2) and are summarized (Table 2).

**TABLE 2:** *In vitro* cardiotoxicity drug screening studies with 3D human engineered heart tissues**.**

Construct (species and cells origin)	Scaffold material	Compounds tested	Readouts	References
3D printed spheroid array (human induced pluripotent stem cells-derived CMs)	Scaffold-free	Blebbistatin, Doxorubicin, Isoproterenol, Propranolol.	Contraction amplitude and kinetics	Arai et al. (2018)
			Cell viability	
Anisotropic cell sheet (human induced pluripotent stem cells-derived CMs)	Scaffold-free	Acetylsalicyclic acid, Cisapride, Flecainide, Procainamide, Terfenadine, Tocainide.	Action potential	Shum et al. (2017)
			Kinetics	
Atrial and ventricular tissue ring (human pluripotent stem cell-derived CMs)	Collagen	Carbamylcholine, Flecainide, Isoproterenol, Lidocaine, Nifedipine, Vernakalant.	Action potential duration	Goldfracht et al. (2020)
			Beat rate	
			Contraction force	
Biowire: Atrial and ventricular tissue strip created by combining CMs and cardiac fibroblasts using a ratio 10:1. They used 6 healthy hPSC lines, 6 iPSC lines from patients with prolonged hypertension, and atrial tissues from 2 healthy hPSC lines.	Collagen and Matrigel	4-AP Carbachol, Diltiazem, Dofetilide, E4031, Isoproterenol, Lidocaine, Milrinone, Nifedipine, Serotonin, Thapsigargin, Verapamil.	Action potential amplitude and kinetics	Zhao et al. (2021)
			Contraction force	
			Ca_2_+ transients	
Atrial tissue strip (CMs generated from human induced pluripotent stem cells)	Fibrin	4-Aminopyridine, Carbachol.	Action potential amplitude and kinetics	Lemme et al. (2018)
Spheroids (monocellular microtissues) Cardiac organoid (50% hiPSC-CMs and 50% non-myocyte (at a 4:2:1 ratio of FBs:HUVECs: hADSCs)	Agarose Hydrogel Micromold	Doxorubicin, JQ1.	RNA sequencing	Richards et al. (2020)
			Transcriptional comparison	
			Contraction force	
			Immunostaining	
			Seahorse assays	
			Calcium imaging	
Cardiac spheroids (human induced pluripotent stem cell lines - SFC086-03-01 and SFC840-03-0)	Laminin	Doxorubicin, Endothelin-1, Acetylsalicylic acid, Isoproterenol, Phenylephrine, and Amiodarone.	Cell number/viability	Christoffersson et al. (2018)
			Presence of Contraction	
			Cell Outgrowth Assay	
3D human cardiac microtissues (composed of CMs and mesenchymal stem cells differentiated from the same hESCs)	Scaffold-free	Aldosterone, Bisphenol A, Metoprolol.	Collagen Deposition	Lee et al. (2022)
			Apoptosis	
			Mitochondrial morphology	
Spheroid (co-culturing human primary or iPSC-derived CMs, endothelial cells and fibroblasts at ratios approximating those present *in vivo*)	Scaffold-free	Doxorubicin, Nitric oxide synthase (NOS) inhibitor L-NIO, and genetic inhibition of endothelial NOS.	Cell viability	Polonchuk et al. (2017)
			Apoptosis	
Spheroid (human induced pluripotent stem cell-derived CMs and human cardiac fibroblasts)	Scaffold-free	4-Aminopyridine, BayK8644, Bisphenol A (BPA), E4031, Isoproterenol, Ranolazine.	Optical mapping of membrane potential	Kofron et al. (2021)
			Immunohistochemistry	
Cardiac Microtissue (Human induced pluripotent stem cell-derived CMs, cardiac endothelial cells, and cardiac fibroblasts)	Scaffold-free	Acyclovir, Amiodarone, Amphotericin B, Bortezomib, Buspirone, Cisapride, Clozapine, Cyclophosphamide, Dasatinib, Donepezil, Doxorubicin, Erlotinib, Fluorouracil, Gemfibrozil, Idarubicin, Imatinib, Isoproterenol, Ketoprofen, Lapatinib, Mebendazole, Methapyrilene, Minoxidil, Mitoxantrone, Naringenin, Nifedipine, Praziquantel, Sorafenib, Sunitinib, Terfenadine.	Cellular viability	Archer et al. (2018)
			Mitochondrial membrane potential	
			Endoplasmic reticulum integrity	
Cardiac organoids (human-induced pluripotent stem cell CMs)	Collagen I and Matrigel	105 compounds	Contraction amplitude	Mills et al. (2019)
			Proliferation	
			Functional effects	
Ventricle-like cardiac organoid chamber (Ventricular CMs generated from the pluripotent human ESC line)	Type-I Bovine Collagen and Matrigel	Digoxin, Disopyramide, Flecainide, Isoproterenol, Nifedipine, Verapamil.	Pressure–volume loops	Li et al. (2020)
			Action potential amplitude and kinetics	
Layered human cells sheet formed from mixed ratio of human-induced pluripotent stem cell-derived CMs, cardiac fibroblasts, and cardiac endothelial cells	Fibronectin and Gelatin	E-4031, Isoproterenol.	FACS qPCR	Takeda et al. (2021)
			Immunostaining	
			Beating rate	
			Contraction kinetics	
Layered human cell sheet of a mixed ratio of human induced pluripotent stem cell-derived CMs, cardiac fibroblasts, and cardiac microvascular endothelial cells	Fibronectin and Gelatin	Chromanol 293B, Dobutamine, E-4031, Flecainide, Glibenclamide, Isoproterenol, Milrinone, Ouabain Pimobendan, Verapamil.	Beating rate	Tadano et al. (2021)
			Contraction kinetics	
			Contractile and relaxation parameters	
			Repolarization parameters	
Microtissue strip (human induced pluripotent stem cell-derived CMs)	Sylgard 184 silicone elastomer molds coated with Pluronic F68 & tissue holders were prepared by cutting nitrocellulose membranes. Scaffold-free.	Ampicillin, E4031, Metformin Hydrochloride, Rosiglitazone, Troglitazone, Erythromycin, Vandetanib, Trovafloxacin, and Tamoxifen.	Contraction amplitude	Lu et al. (2017)
			Beating rate	
			Cell viability	
Microtissue strip (neonatal rat ventricular myocytes or a mixed ration of 93% human-induced pluripotent stem cell CMs and 7% human mesenchymal stem cells)	Collagen I	Carbonyl cyanide m-chlorophenyl hydrazone, Sunitinib, Staurosporine.	Apoptosis	Truitt et al. (2018)
			Beating rate	
			Mitochondrial membrane potential	
			ATP assay	
Human ventricular-like cardiac tissue strips and organoid chambers - (human pluripotent stem cell-derived CMs)	Collagen I and Matrigel	Acetylsalicylic Acid, Amitriptyline, Bepridil, Caffeine, Digoxin, Disopyramide, Dobutamine, Dopamine, Flecainide, Glibenclamide, Isoproterenol, Levosimendan, Lidocaine, Lisinopril, Mibefradil, Milrinone, Nifedipine, Norepinephrine, Pravastatin, Procainamide, Quinidine, Ramipril, Tocainide, Tolbutamide, Verapamil.	Contraction force	Keung et al. (2019)
			Pressure-Volume Loops	
Tissue strip (human induced pluripotent stem cell derived CMs)	Matrigel and Fibrin	Isoproterenol, Verapamil.	Beat Rate	Bielawski et al. (2016)
			Twitch force	
Tissue strip (Induced pluripotent stem cell derived CMs originated from rat, mouse, and human)	Matrigel and Fibrin	E4031	Contraction force	Mannhardt et al. (2017)
			Contraction kinetics	
Tissue strip (human induced pluripotent stem cell-derived CMs)	Matrigel and Fibrin	BayK-8644, Digoxin, EMD-57033, Isoprenaline, Nifedipine, Ryanodine, Thapsigargin.	Contraction force Contraction kinetics	Mannhardt et al. (2020)
Tissue strip (human induced pluripotent stem cells)	Fibrin	Caffeine, Isoproterenol, Nifedipine, Thapsigargin, Verapamil	Amplitude and frequency of contractions, calcium handling, force generation, excitation threshold and maximum capture rate, cell and tissue morphology, ultrastructure, gene expression and immunohistochemistry	Ronaldson-Bouchard et al. (2018)
Tissue strip (human-induced pluripotent stem cell-derived CMs)	Fibrin and Matrigel	Acetylsalicylic acid, Atenolol, Captopril, Citalopram, Clonidine, Dobutamine, Doxorubicin, Enalaprilat, Epinephrine, Flecainide, Forskolin, Glibenclamide, Itraconazole, Ivabradine, Levosimendan, Milrinone, Omecamtiv, Paracetamol, Phentolamine, Pimobendan, Pravastatin, Sildenafil, Sorafenib, Sunitinib, Terbutaline, Tolbutamide, Verapami,l Zimelidine.	Contraction amplitude and kinetics	Saleem et al. (2020)
Biowire Organ-on-a-chip (human induced pluripotent stem cell -derived CMs and human ventricular fibroblasts)	Fibrin and Matrigel	Angiotensin II, Losartan, Relaxin, Saracatinib.	Contraction force and kinetics	Wang et al. (2021a)
			Beat rate	
			Ca2+ transients	
			Cell viability	

hPSC-derived organoids are versatile tools for drug screening. For example, hPSC-derived heart organoids have been used to screen for compounds that promote cardiac growth while minimizing impacts on heart rhythm and contractility (Miyamoto et al., 2021; Cho et al., 2022). Furthermore, organoid-on-a-chip platforms have opened new opportunities for investigating complex interactions between multiple tissue types. One cardiac organoid-on-a-chip platform demonstrated differentiation of hPSCs into distinct myocardial, endothelial, and epithelial layers (Hofbauer et al., 2021). Another model generated cardiac organoids with chambers, epicardium, myocardium, and regional organization of ventricular and atrial CMs (Lee et al., 2022). These 3D organoid drug screening platforms can eliminate false positives detected in traditional 2D cultures, predict effects on cardiac contractility and rhythm, and identify key regulatory pathways driving human CM proliferation, like the mevalonate pathway (Mills et al., 2017; 2019). Moreover, Kofron and Mende (2017) presented a 3D human cardiac microtissue model composed of hPSC-derived CMs and human cardiac fibroblasts that differentiates between high-risk and low-risk drugs by detecting changes in action potentials.

*Cardiotoxicity screening.* To ensure drug safety, it is crucial to evaluate cardiotoxicity, especially for patients with pre-existing CVDs (Stone et al., 2021). Evaluating cardiotoxicity amongst certain drug classes (see Table 1) can help healthcare providers select the proper medications for their patients. While determining toxicity profiles during pharmaceutical development is costly and requires animal studies and clinical trials, early detection can help companies make better decisions and reduce development costs and time. One of the first studies utilizing hPSC-CM platforms for cardiotoxicity testing set up an *in vitro* cardiac safety index (CSI) for 21 small molecules in the tyrosine kinase inhibitor drug class using cell survival and contractility assays (Sharma et al., 2018). The *in vitro* CSI was consistent with the small molecules’ current clinical cardiotoxicity profiles. Furthermore, since the onset of the COVID-19 pandemic, many publications have shown how 2D hPSC-CMs could be used for cardiotoxicity studies. A recent review highlighted heart complications associated with long-term chloroquine or hydroxychloroquine (HCQ) use, leading to concerns about its potential cardiotoxicity in the treatment of COVID-19 (Kamp et al., 2020). HCQ has been linked to the development of torsade de pointes (TdP) and other arrhythmias, while other COVID-19 drugs have low proarrhythmic risk. To assess cardiotoxicity caused by COVID-19 treatments in nonclinical settings, 2D hPSC-CMs were shown to be a useful tool (Yanagida et al., 2021). The proven efficacy of 2D hPSC heart models has given credence to efforts to make use of more complex 3D hPSC-derived heart tissues. For example, Archer *et al.* (2018) evaluated drug-induced changes in mitochondrial membrane potential, endoplasmic reticulum integrity, and cellular viability of 15 FDA-approved structural and 14 nonstructural cardiotoxins on 3D microtissues containing hPSC-CMs, endocardial cells, and cardiac fibroblasts. This study showed a high-throughput approach with 73% sensitivity and 86% specificity in predicting *in vivo* outcomes of structural cardiotoxins. Moreover, Tadano *et al.* (2021) developed a 3D vascularized cardiac tissue model using hPSC-derived CMs and fibroblasts for the assessment of cardiotoxicity, and the expected effects of several drugs with different mechanisms of action were verified by cell motion imaging. Furthermore, unlike direct-assembled hPSC-derived heart tissues using CTE methods, self-assembled cardiac organoids have been used to quantify drug effects on cardiac differentiation, contractility, and morphology to study drug-induced cardiotoxicity during development (Hoang et al., 2021). This platform can be used for pharmaceutical development and fetal safety assessments. Furthermore, Polonchuk *et al.* (2017) showed that administration of doxorubicin to cardiac spheroids increased cellular apoptosis and the cardiotoxic effects of doxorubicin could be diminished by co-administration with L-NIO, a nitric oxide synthase inhibitor. Cardiac organoids have also been used to simulate pre-existing cardiovascular conditions to study doxorubicin-induced cardiotoxicity (Richards et al., 2020). Richards *et al.* demonstrated that doxorubicin administered in culture with their cardiac organoid model reduced contraction amplitude and increased smooth muscle actin disorganization in both control and infarcted organoids. The multi-well format of 3D EHTs is also useful for high throughput pharmaceutical testing. Christoffersson *et al.* (2018) conducted a study to evaluate the potential of a high throughput cardiac cell outgrowth assay using cardiac spheroids on a microfluidic device. Researchers tested six compounds at three concentrations for 48 h and used non-invasive image-based readouts to assess the spheroid quality. Furthermore, Sallam *et al.* (2022) recently used a hPSC-derived cardiac organoid model to examine the cardiovascular effects of two immunosuppressant drugs: tacrolimus and sirolimus. Sirolimus was found to limit fibrosis more so than tacrolimus in cardiac multicellular clusters, suggesting its potential for limiting adverse remodeling in CHF patients. Overall, the hope is that in the future 3D heart models can be used to simulate preclinical trials *in vitro* with patient-derived cell lines to predict clinical toxicity profiles. Generating a standardized CSI for available medications with 3D hPSC-human heart models can help identify the cardiotoxic effects of novel drugs by comparing their CSI to that of existing drugs. This approach would be useful for evaluating the safety of new drugs for patients. In summary, multiple studies, some of which are reviewed here, support the use of 3D hPSC-human heart platforms to efficiently investigate drug-induced cardiotoxicity during early-stage drug development.

## Challenges and limitations

Considerable progress has been made in developing models of 3D hPSC-derived heart tissue for use in drug/cardiotoxicity screening and disease modeling. However, the forces generated by 3D hPSC-heart models are much smaller than those of native hearts due to the fetal/neonatal resemblance of hPSC-CMs. Tissue maturation and electrical/mechanical stimulation are potential solutions to this problem, but optimal stimulation protocols have yet to be standardized (Karbassi et al., 2020). Increasing the complexity and heterogeneity of current 3D hPSC-heart models, as well as developing methods for vascularization and perfusion, can enhance their functionality and better mimic native heart tissue (Clevers, 2016). Additional goals include improving scalability, maturity, and functional capacity. Precision medicine approaches can be developed for individual cardiomyopathies and the repurposing of existing drugs through functional and molecular phenotyping in human cardiac samples. Although 3D tissue cultures present challenges, such as poor vascularization, handling difficulties, and lack of standards for efficacy and toxicity testing, strategies are being developed to address them, such as the creation of a 3D microchannel scaffold and perfusable microvascular constructs (Takebe et al., 2017). Regarding organoids, one limitation is that they often have multiple mis-assembled regions, which can affect reproducibility. According to a review published in Nature, organoids with chromosomal instability or mismatch-repair deficiency continuously gain chromosome mis-segregations or replication errors during culture, and researchers should keep in mind the impact of long-term culture on the genomic architecture of tumor organoids (Fujii and Sato, 2021). Monitoring genetic stability by karyotyping and expansion from tested lines can be done to ensure genomic instability in assays (Li et al., 2020). Modeling other patient specific differences, such as ethnicity and sex, is also of interest. One of the challenges in hPSC research is the limited representation of different ethnic groups in available cell lines, which limits the potential for studying the genetic and epigenetic factors that influence cell differentiation and disease susceptibility (Laurent et al., 2010; Tofoli et al., 2016; Ghosh et al., 2022). To address this gap, several initiatives have been launched to generate and characterize hPSC lines from diverse populations, such as the Human Heredity and Health in Africa (H3Africa) project (Matimba et al., 2018). These efforts aim to increase the diversity and quality of hPSC resources and to facilitate their availability among researchers worldwide. Sex differences can also play a critical role in modulating cardiac physiology and pathology in humans (Gerdts and Regitz-Zagrosek, 2019; Lock et al., 2022). Sex hormones can affect the expression of genes, proteins, and ion channels in CMs and have impacts on the extracellular matrix, inflammatory response, and angiogenesis (Viloria-Petit et al., 2013). Most cardiac organoids are cultured in hormone-free media, which does not reflect the physiological or pathological conditions of males and females. To address this issue, some studies have used hormones to mimic fluctuations during the menstrual cycle or menopause. Lock *et al.* (2022) developed a framework for developing sex-specific engineered heart models using hormone-responsive hydrogels that can release estrogen or testosterone in response to external stimuli. They found that hormone fluctuations can affect contractility, calcium handling, and electrophysiology of cardiac organoids derived from male or female iPSCs. Similarly, Sim *et al.* (2021) exposed cardiac organoids to varying types of hormones, such as estradiol, progesterone, dihydrotestosterone, and testosterone, and revealed diverse and sometimes contradictory effects on structure and function. It is also worth noting that in the context of cardiotoxicity studies, there is data indicating that females may be more susceptible cardiotoxic side effects of drugs like doxorubicin than males (Moulin et al., 2015). By incorporating sex hormones or using cell lines from both sexes, we can understand mechanisms underlying sex differences in cardiac physiology and pathology to develop more effective drugs for both men and women. Establishing standards for each target disease or drug response is also crucial for the future of CTE research. Other future directions for human CTE include using injectable hydrogels and utilizing the elastic modulus of neonatal and adult heart tissue as a basis for selecting proper materials. Advancements in understanding the mechanisms of atherogenesis, cell adhesion, and cell biomechanics are also crucial for cardiovascular tissue engineering. Ongoing research and development will be necessary to overcome current challenges and set up the field’s clinical readiness.

## Conclusion

Drug-induced toxicity is a crucial aspect of pharmaceutical development and patient care, particularly cardiotoxicity resulting from off-target drug effects during chemotherapy (Guengerich, 2011). This complication is a significant cause of drug withdrawals. Detecting drug-induced cardiotoxicity in humans is complex due to slow manifestation and patient-specific variations (Cameron et al., 2016; Herrmann et al., 2016). Animal models have been used for drug screening, but species differences limit their translational and clinical relevance (Bailey et al., 2013; 2014). To address this challenge, iPSC technology can be integrated with state-of-the-art tools such as 3D printed microfluidic chips, CRISPR-based gene editing, and machine learning to establish a scalable and personalized drug testing platform. While PSC organ models are still in their infancy with limitations in consistency, maturity, and physiological relevance, continued interdisciplinary research can create a promising tool for cardiotoxicity screening in the future. Two critical factors will need to be considered when selecting an *in vitro* platform for cardiotoxicity and drug screening: the model’s ability to closely replicate physiological relevance and scalability. Organoids and microfluidic chips are more relevant than 2D models or spheroids in replicating physiological relevance (Duval et al., 2017). However, 2D models are commonly used in the initial stages of drug screening despite their limited physiological relevance. A rational strategy would be to use 2D models for preliminary drug screening before exploring the more accurate and comprehensive understanding of drug effects offered by 3D organoids or microfluidic platforms. Recent advancements in biomaterial technologies, including iPSC-based drug testing (Rowe and Daley, 2019), 3D printing with iPSC-derived cells (Maiullari et al., 2018; Shukla et al., 2022), single-cell RNA sequencing (Jovic et al., 2022), CRISPR-based gene editing with patient-specific iPSCs (Jehuda et al., 2018; Paik et al., 2020), and machine learning with big data (Vo et al., 2020; Tripathi et al., 2021), can significantly expand the scope of cardiotoxicity and drug screening with 3D human heart organoid platforms.
